# Electrochemical Sensor Based on Poly(Azure B)-DNA Composite for Doxorubicin Determination

**DOI:** 10.3390/s19092085

**Published:** 2019-05-05

**Authors:** Anna Porfireva, Vyatseslav Vorobev, Sofya Babkina, Gennady Evtugyn

**Affiliations:** 1 A.M. Butlerov’ Chemistry Institute of Kazan Federal University, 18 Kremlevskaya Street, Kazan 420008, Russia; porfireva-a@inbox.ru; 2 Interdisciplinary Center of Analytical Microscopy of Kazan Federal University, 18 Kremlevskaya Street, Kazan 420008, Russia; slavik.ksu@mail.ru; 3 Analytical Chemistry Department of the Lomonosov’ Institute of Fine Chemical Technologies, MIREA—Russian Technological University, 86 Vernadsky Prospect, Moscow 119571, Russia; sofya.babkina@gmail.com

**Keywords:** DNA sensor, doxorubicin determination, voltammetric sensor, electropolymerization, Azure B

## Abstract

A new voltammetric DNA sensor has been developed for doxorubicin determination on the platform of a glassy carbon electrode (GCE) covered with electropolymerized Azure B film and physically adsorbed native DNA. The redox properties of polymeric Azure B were monitored at various pH and scan rates. DNA application decreased the peak currents related to polymeric and monomeric forms of the dye, whereas incubation in doxorubicin solution partially restored the peaks in accordance with the drug and DNA concentration. The relative shift of the cathodic peak current caused by doxorubicin depended on the nominal DNA concentration and its application mode. In optimal conditions, the DNA sensor makes it possible to determine between 0.1 μM to 0.1 nM doxorubicin (limit of detection 7 × 10^−11^ M). The DNA sensor was tested on commercial doxorubicin formulations and on artificial samples the mimicked electrolyte content of human serum.

## 1. Introduction

DNA sensors have found increasing attention during the past decade for the fast and sensitive determination of complementary sequences such as hybridization events [1] and antitumor drugs [2,3], as well as for the detection of DNA damage caused by physical [4] and chemical [5,6,7] factors. The interest in DNA sensors has increased due to its advantages, including compatibility with conventional measurement equipment, low-cost, possibility to use for point-of-care diagnostics, in-flow analysis mode, and intuitive and understandable design and data interpretation.

Despite the obvious progress in DNA sensor design, there are problems that prevent their wider application in medicine. DNA sensors are related to the complicated assembling of biorecognition layers and insufficient sensitivity of analyte detection. This is particularly true for the determination of cytostatic drugs that prevent DNA transcription in cancer cells [8] and are frequently used in cancer chemotherapy. Although rather effective, anticancer drugs are also toxic; the gap between therapeutic and toxic doses is narrow and can be surmounted for individual patients with high sensitivity toward certain drugs. This can be fully referred to by anthracycline preparations, i.e., doxorubicin, daunorubicin, epirubicin, and idarubicin that are applied in the treatment of solid tumors and leukemia [9] while also having cardiotoxic side effects [10]. For this reason, great efforts have been directed to search for safer and more efficient representatives of this class of drugs.

The detection of DNA interactions with low-molecular compounds, including the drugs mentioned above, is mainly based on the optic methods of analysis (e.g., UV-vis [11] and fluorescence [12] spectroscopy, circular dichroism [13], and surface plasmon resonance [14]). They can be combined with chromatography or electrophoresis for separation and combined with DNA for the concentration of the analyte molecules and their adducts. Being sufficiently sensitive and selective, conventional analytical instrumentation is less appropriate for application in portable measurement devices. Its application assumes high qualification of personnel and often time needs labor-consuming sample treatment. Besides, optic detection has limitations in working with colored, turbid, and inhomogeneous samples.

DNA sensors with electrochemical transduction systems show undisputable advantages over the conventional techniques described above due to high sensitivity of the signal, universal approaches to biochemical recognition detection, compatibility with common measurement equipment, well elaborated theory, cheap design, low specific requirements to the quality of labor staff, and simple sample treatment protocols [15]. 

DNA-drug interactions can be electrochemically recorded following three different approaches [2,5]: (1) direct and mediated oxidation of guanine and adenine nucleobases in the DNA sequence on the electrode [16]; (2) monitoring of the redox activity of the drugs accumulated on immobilized DNA [17,18]; and (3) recording changes in mobility or redox activity of specific probes added together or after an analyte to the working solution [19]. 

In the latter case, DNA molecules are often implemented in the polymeric matrix that provides both mechanical support and electric wiring of biopolymer molecules to the electrode. Polyaniline, a conjugated linear polymer of aniline, is mostly used in such sensors due to its intrinsic electroconductivity and compatibility of charge distribution with that of phosphate residues in the DNA chain [20,21]. Similar behavior exerts polypyrrole, though its response to biochemical reactions with DNA is milder than that of polyaniline [22]. Appropriate DNA sensors were reported for the determination of anthracyclines [23,24,25,26], salicylates [22], isoproterenol [27], and phenothiazine drugs [18,28]. However, electroconductivity of polyaniline is observed in strongly acidic conditions. In neutral and basic media, it is neither conductive nor redox active. Its necessity in strong acids during the step of biosensor assembly might result in partial denaturing DNA and therefore decrease the affinity of DNA-drug interactions. Besides, many other compounds like antioxidants and reactive oxygen species can both affect polymer redox properties and DNA response toward specific factors.

To some extent, drawbacks of polyaniline as the DNA support in the biosensor assembly can be overcome by the application of other polymerizable compounds with intrinsic redox activity, e.g., thiazine and phenazine dyes (Methylene blue [29], Methylene green [30], and Neutral red [31,32]). However, changes in their redox activity caused by DNA interactions are mostly insufficient for detection of small drug molecules. Meanwhile, DNA sensors based on redox active dyes were successfully used for the detection of oxidative DNA damage and aptamer-analyte interactions by shifts of the peak currents and potentials, as well as by changes in the electrochemical impedance parameters [29,30,31,32]. 

Azure B (1) is another representative of the thiazine dye family, which differs from those mentioned above by a higher number of methyl radicals at aromatic amino groups of phenothiazine core. Steric loading of aromatic systems partially prevents the reactions of oxidative oligomerization and redox activity of the products. Low solubility and self-aggregation of the monomeric dye makes its electrochemical properties and polymerization efficiency sensitive to working conditions and electrode materials. Electrodes modified with carbonaceous materials bearing poly(Azure B) have been successfully used for determination of nicotinamide adenine dinucleotide (NADH) [33,34], hydroquinone [35], and in the assembly of the enzyme sensor with immobilized tyrosinase [36]. Recently, growth of the poly(Azure B) film was monitored by cyclic voltammetry and chronoamperometry on polycrystalline Au electrode [37]. However, to the best of our knowledge, poly(Azure B) has never been used in the assembly of DNA sensors. In this work, we report for the first-time the application of a DNA sensor with electropolymerized Azure B film for the detection of DNA-drug interactions. 

## 2. Materials and Methods

### 2.1. Reagents

Doxorubicin (1), Azure B, HEPES (4-(2-hydroxyethyl)-1-piperazineethanesulfonic acid), glucose, mannose, mannitol, bovine serum albumin (BSA), and ascorbic and uric acids were purchased from Sigma-Aldrich (USA). Low-molecular DNA was procured from salmon sperm (Cat. No. 31149, average mol. mass 4.6 kDa [38]) and purchased from Sigma-Aldrich (USA). Other reagents were of analytical grade. Deionized Millipore^®^ water was used for the preparation of working solutions. The pH dependence of the poly(Azure B) redox properties was monitored using 0.05 M glycine buffer (pH 3.0), 0.05 M acetate buffer (pH 4.0 and 5.0), 0.025 M phosphate buffer containing 0.1 M NaNO_3_ (pH 6.0), and 0.1 M HEPES with 0.03 M NaCl (pH 7.0 and 8.0).

Doxorubicin-LANS^®^ (“Verofarm”, Russia) and Doxorubicin-TEVA^®^ (“TEVA Pharmaceutical Industries Ltd.”, the Netherlands) were purchased in the local market. 


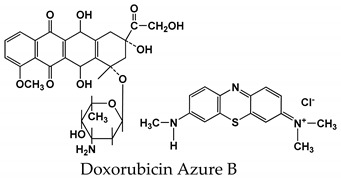
(1)

### 2.2. Apparatus

Voltammetric measurements were performed at room temperature with AUTOLAB PGSTAT 302N (Metrohm Autolab, B.V., the Netherlands) equipped with a FRA2 module for electrochemical impedance spectroscopy (EIS) measurements. The DNA sensor was assembled on a glassy carbon electrode (GCE, 1.67 mm^2^). Platinum wire was used as an auxiliary electrode and Ag/AgCl/3 M KCl as a reference electrode (Metrohm Autolab). The EIS spectra were recorded with the amplitude of the applied sine potential of 5 mV; frequency varied in the range from 100 kHz to 0.04 Hz. The capacitance and the charge transfer resistance were calculated from the Nyquist diagram for Randles equivalent circuit using NOVA software (Metrohm Autolab).

The scanning electron microscopy (SEM) images were obtained with high-resolution field emission scanning electron microscope Merlin™ (Carl Zeiss).

### 2.3. DNA Sensor Preparation

Prior to use, the GCE was mechanically polished with the silica polishing kit (CH Instruments, Inc., Cat. No CHI120), rinsed with deionized water, ethanol, and acetone. Afterward, it was immersed in the electrochemical cell with 5 mL of 0.1 M HEPES, pH 7.0, containing 0.03 M NaCl (HEPES–NaCl buffer). After equilibration, 150 μL of 1.03 mg/mL aqueous Azure B solution were added. Full dissolution and preparation of standard dye solution requires sonication. The electrode was polarized at 1.0 V for 300 s and then 40 cycles of the potential from −0.4 to 1.1 V were run with the scan rate of 50 mV/s. Next, the electrode was moved to the HEPES–NaCl buffer and 10 cycles of the potential between −0.5 and 0.5 V were made to stabilize the currents recorded.

The DNA immobilization was performed by applying 2 μL of 1 mg/mL DNA solution onto the poly(Azure B) film. Next, the wetted surface was either dried at room temperature or capped with a plastic tube for 20 min. In the latter case, a full drying of the solution was prevented to control DNA quantities adsorbed on the polymer film. In both cases, the DNA sensor was finally washed multiple times with deionized water and transferred onto the working cell with 5 mL of HEPES–NaCl solution.

### 2.4. Doxorubicin Measurements and Real Sample Assay

Prior to deposition on the poly(Azure B) film, a series of DNA standard solutions was prepared by mixing stock solution with aqueous doxorubicin solution or HEPES–NaCl in the 1:1 ratio (v:v) to reach its concentration range from 1.0 to 1 × 10^−6^ mg/mL. The final concentration of doxorubicin varied from 1 × 10^−4^ to 1 × 10^−11^ M. Then the mixture was casted on the electrode surface and left for 20 min. Prior to signal measurement, the DNA sensor was washed several times with deionized water. Spiked plasma samples were prepared from Ringer-Locke’s solution (9.00 g/L NaCl, 0.42 g/L KCl, 0.25 g/L CaCl_2_, 0.30 g/L MgSO_4_, 0.50 g/L NaH_2_PO_4_ × 2H_2_O, and 1.0 g/L glucose, pH 5.94) imitated blood plasma ionic content. The doxorubicin-LANS^®^ and Doxorubicin-Teva^®^ preparations were first dissolved in 0.9% NaCl solution and then diluted to the working concentration with HEPES–NaCl. In a similar manner, standard solutions of doxorubicin containing 4.0–40 mg/mL BSA were prepared and used for interferences investigation.

Blood serum taken from healthy volunteers was treated as follows: 1 mL of the plasma sample was mixed with 1 mL of 0.1 M HEPES–NaCl buffer and 1 mL of methanol. The mixture was vortexed and then centrifuged at 1000 *g* for 10 min. The supernatant was filtered and mixed with methanol stock solution of doxorubicin to its final concentration of 50 nM. Prior to contact with the DNA sensor, it was diluted with HEPES–NaCl solution into a final volume of organic solvent not more than 5%. A measurement of doxorubicin concentration was performed as described above for artificial plasma samples. All the assays were performed in triplicate.

## 3. Results and Discussion

### 3.1. Azure B Polymerization and Redox Properties of Poly(Azure B) Layer

**Figure 1:**
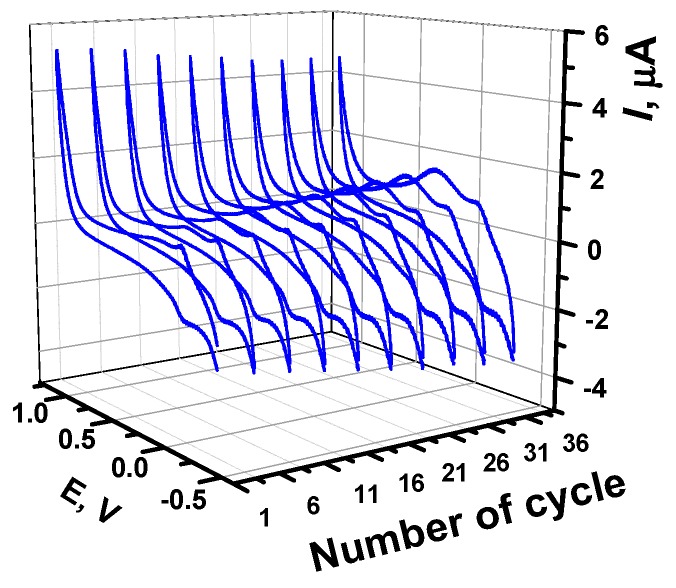
Cyclic voltammograms recorded on glassy carbon electrode (GCE) in 0.1 M HEPES (4-(2-hydroxyethyl)-1-piperazineethanesulfonic acid) containing 0.03 M NaCl, pH 6.9, 0.1 mM Azure B in the potential range from −0.4 to 1.1 V. 40 cycles were recorded with the scan rate 50 mV/s, each fifth cycle is shown.

Azure B belongs to the class of phenothiazine dyes that are able to electropolymerization via the oxidation of a primary cationic form to di-cation, which is then coupled to the initial molecule of monomer and initiated in the formation of polymeric products. The electropolymerization of Azure B on GCE by using continuous cycling of the potential resulted in consecutive increases of the peak currents related to both monomeric and polymeric forms of the dye (Figure 1). The choice of HEPES buffer is related to poor solubility of Azure B and its inclination to self-aggregation, which decreases the reproducibility of the cyclic voltammograms and efficiency of polymerization.

Preliminary polarization of the GCE at 1.0 V for 300 s improved polymerization and increased appropriate peak currents on voltammogram. Polymerization was accelerated with increasing number of potential cycles.

**Figure 2:**
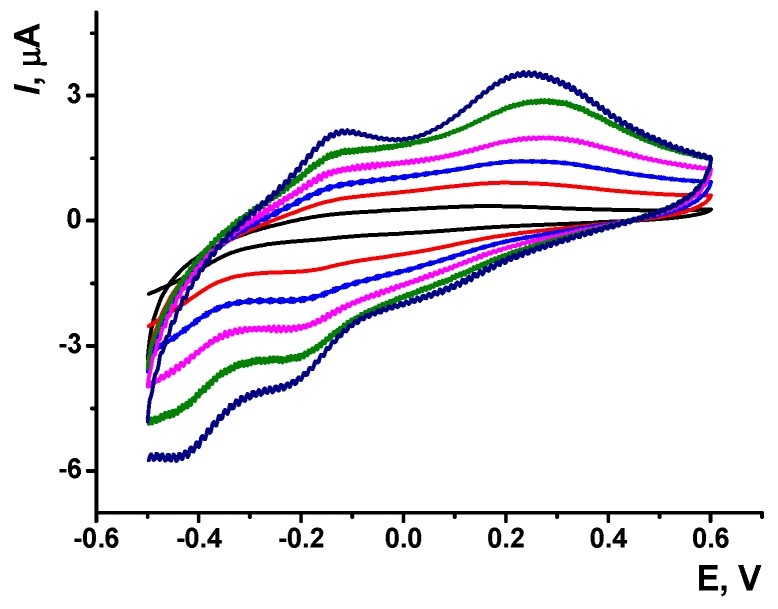
The dependence of the poly(Azure B) peak currents on the potential scan rate. Cyclic voltammograms recorded on GCE modified with poly(Azure B) in 0.1 M HEPES containing 0.03 M NaCl, pH 6.9, scan rate 10, 30, 50, 70, 90, and 100 mV/s.

Three peaks—the cathodic peak at −0.18 V and two anodic peaks at −0.09 and 0.25 V—appear on voltammograms after polymerization (Figure 2). The position and relative height of the peaks were similar to those previously reported for GCE [39] and Au [37]. In the latter case, adsorption of monomeric Azure B in the polymer film was confirmed using the electron dispersive X-ray analysis. The peak pair observed at −0.18 and −0.25 V was attributed to the redox conversion of the monomers entrapped in the polymer film, whereas the new anodic peak that appeared after the first several cycles at about 0.25 V and gradually shifted to higher anodic potential corresponds to the polymeric form of the dye. Corresponding cathodic peak appeared first as a shoulder, at about 0.19 V, and then as a wave in the final cycles of the potential.

A sharp increase of the anodic current near 1 V (see Figure 1) is related to the oxidation of the monomer to di-cation that initiates polymerization. If the reverse potential was below 1.0 V, changes in the voltammograms with increased number of cycles were much milder. Previously, the stability of the poly(Azure B) at high anodic potentials (about 1.0 V) was found to be quite sufficient [37].

The resulting poly(Azure B) film was stable in repeating cycling of the potential performed in the absence of the monomer. The peaks mentioned were stabilized to third cycle and remained constant during at least one weak operation in HEPES–NaCl solution.

A variation of the scan rate (ν) (Figure 2) indicated the limitations of the electrode transfer by the surface confined processes. In double logarithmic coordinates of peak currents *I_p_* on scan rate the slope of the curves was near 1.0 (Table 1).

The electron transfer coefficient (α) was calculated using Laviron’s theory and International Union of Pure and Applied Chemistry (IUPAC) recommendations [40] from the dependence of the peak potential *E_p_* on the scan rate in accordance with Equation (2)

(2) $$\begin{array}{c} E_{pc}=E^{0\prime }+\frac{RT}{\alpha nF}\ln\frac{RTk_{et}}{\alpha nF}-\frac{RT}{\alpha nF}\ln v\\ E_{pa}=E^{0\prime }+\frac{RT}{(1-\alpha )nF}\ln\frac{(1-\alpha )nF}{RTk_{et}}+\frac{RT}{(1-\alpha )nF}\ln v \end{array}$$

Here, *E_pa_* and *E_pc_* are the potentials of cathodic and anodic peak currents, *E*^0′^ is formal redox potential, *R* is universal gas constant, *T* is absolute temperature, *n* is the number of electrons transferred in limiting step of the reaction, *F* is Faraday constant, and *k_et_* is the electron transfer rate. For monomer oxidation, α = 0.44. This indicates a symmetrical transient state of electron transfer; no significant changes in the polymer structure were caused by the reverse oxidation-reduction cycle. Such conditions make the poly(Azure B) attractive for the use in DNA sensor assembly because reversible changes in the redox status of the surface should be sensitive to the changes in the electrostatic interactions with DNA participation and hence to specific DNA interactions.

**Figure 3:**
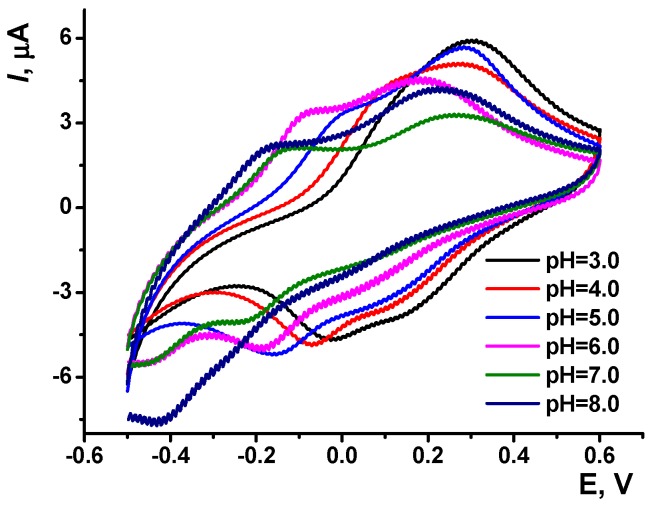
The pH dependence of the poly(Azure B) electrochemical behavior. Cyclic voltammograms recorded at various pH values on GCE modified with poly(Azure B), scan rate 100 mV/s.

The changes in cyclic voltammograms obtained at various pH values are illustrated in Figure 3.

Poorly resolved in acidic media, two anodic peaks became better recognized starting from pH 5.0. In the whole pH range considered, cathodic peaks were less pronounced against anodic peaks. Resolution of cathodic peaks becomes worse when the pH increased to 8.0.

The anodic peak current attributed to the monomeric, which depended on the pH in acidic media with a slope of 29.9 ± 0.4 mV/pH (pH = 3.0–6.0). This value corresponded to the ratio of H^+^ ions and electrons transferred in a rate determining step equal to 1:2. In the literature [33,34], two electrons and two protons are transferred at pH < 7.0. The contradiction is likely related to the thinner film obtained on GCE in this work against that on electrodes modified with carbon materials with a higher specific surface. In thick films, H^+^ exchange can be limited by own buffering properties of the polymer. Similar effect can be expected from surface carboxylate groups of carbonaceous modifiers.

The stoichiometry of redox reaction assumes changes in the charge of the Azure B layer. The cathodic peak currents had smoothened shape with weakly resolved maxima and their pH dependence was similar to that of anodic peaks.

### 3.2. DNA Deposition and Determination

Two protocols have been used for electrostatic adsorption of the DNA molecules. In the first one, GCE covered with the poly(Azure B) film was incubated in the DNA solution and then washed with deionized water. In the second protocol, a certain aliquot of the DNA solution was left drying on the working surface of the electrode. After that, the electrode was washed with deionized water and an HEPES–NaCl buffer. In both cases, DNA sensors were stored in dry conditions, if not used.

Assembling the DNA layer onto the electrode was monitored using EIS in the presence of ferricyanide redox probe [Fe(CN)_6_]^3−/4−^. The Randles equivalent circuit was used for EIS data fitting. The roughness parameter *n* was equal to 0.92–0.95, indicating that the constant phase element behaves as capacitance. Indices correspond to the electrode-polymer and polymer-solution interfaces.

**Figure 4:**
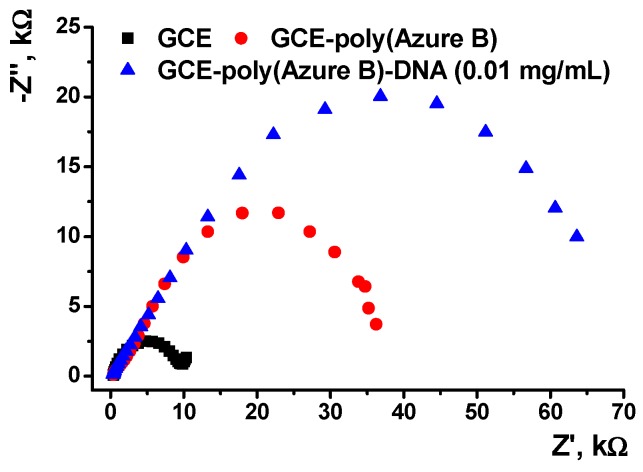
The electrochemical impedance spectroscopy (EIS) characterization of the surface layer assembling. The Nyquist diagram of impedance spectra recorded on GCE covered with poly(Azure B) (40 cycles) and incubated in 0.01 mg/mL DNA solution. Measurements in the presence of 0.01 M K_3_[Fe(CN)_6_] and 0.01 M K_4_[Fe(CN)_6_] at 0.27V. Frequency range 0.04 Hz–100 kHz, amplitude 5 mV.

The Nyquist diagrams are presented in Figure 4. Semicircle in the area of high frequencies corresponded to the charge transfer limitation. Its diameter is equal to the charge transfer resistance, which consecutively increased from 8.0 (bare GCE) to 35 and 68 kΩ (poly(Azure B) prior to and after DNA deposition, respectively. Increase in the charge transfer resistance is due to implementation of non-conductive DNA molecules in the surface film.

**Figure 5:**
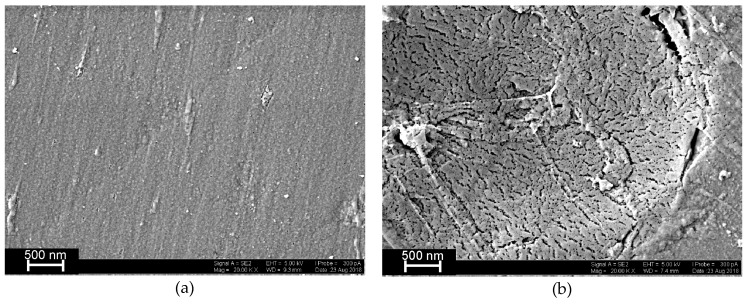
Scanning electron microscopy (SEM) images of the GCE covered (**a**) with poly(Azure B), 40 cycles of electropolymerization, and (**b**) DNA deposited for 20 min from 0.1 mg/mL solution.

The SEM images (Figure 5) confirm deposition of the DNA molecules onto the poly(Azure B) layer due to electrostatic adsorption.

In the presence of DNA, the peak currents of poly(Azure B) decreased in a degree dependent on the incubation protocol and the DNA concentration. Changes are more pronounced for the cathodic peak used in all the following measurements.

Maximal shift of the cathodic peak current caused by the DNA deposition was equal to 50% of its initial value for incubation protocol and 70% for drying-washing protocol of the DNA deposition. In an appropriate blank experiment, HEPES, NaCl, and deionized water were used instead of the DNA solution and 2.5% deviation of the peak currents was observed for six measurements with individual sensors. The difference in the variation of the peak current observed for two protocols can be due to the DNA aggregation on the stage of electrode drying. Aggregates formed can partially leave the surface in washing steps. Thus, real surface of the polymer film covered with DNA becomes higher for the poly(Azure B) film adsorbing the DNA molecules from their solution without its drying. In the next experiment, incubation of the electrode in the DNA solution without its full drying was chosen.

The shift of the poly(Azure B) peak current linearly depended on the DNA concentration within two ranges (3):

1 × 10^−6^–0.2 mg/mL: *I*/*I*_0_, % = (69.2 ± 0.4) − (2.77 ± 0.11) × log(*c_DNA_*, mg/mL), *R*_2_ = 0.9891, *n* = 7 0.2–1.0 mg/mL: *I*/*I*_0_, % = (59.6 ± 0.7) − (15.7 ± 0.4) × log(*c_DNA_*, mg/mL), *R*^2^ = 0.9562, *n* = 5 (3)

Here, *I*_0_ and *I* are cathodic peak currents measured prior to and after the contact of the sensor with DNA. Considering the aliquot volume (2 μL), the electrochemical sensor developed makes it possible to detect 0.43 femtomole of DNA. Meanwhile, the low slope of the dependence is comparable with the relative deviation of the signal and makes the measurement semi-quantitative, allowing its establishing to be the only order of magnitude of the DNA content.

Two linear pieces of the calibration curve can be explained by different driving forces of the DNA deposition on the electrode surface. At low concentrations, DNA can be preferably attached to cationic sites of Azure B, whereas higher DNA quantities after saturation of the polymer surface are adsorbed on the electrode. The latter process decreases the real surface of electrode involved in the electron exchange and hence the slope of the second linear piece of calibration curve has a much higher slope than that obtained with lower DNA quantities. It should be mentioned that the application of BSA in concentrations below 40 mg/mL did not regularly affect the voltammograms recorded though increased deviation of the curves. Although further investigation of selectivity calls for more experiments, it can be concluded that serum proteins that accompany drugs in blood serum do not influence electrochemical response of poly(Azure B) film.

### 3.3. Doxorubicin Determination

Doxorubicin belongs to the family of cytostatic drugs applied in chemotherapy of breast and bladder cancer, Kaposi’s sarcoma, lymphoma, and acute lymphocytic leukemia [41,42]. Being approved for medical application in 1974, it remains one of the more frequently used preparations applied alone and in various mixtures. However, its clinical activity is limited by acute cardiotoxicity [43]. Presently, doxorubicin is determined by liquid chromatography [44] and electrophoresis [45]. Being sensitive and selective, such conventional techniques cannot provide fast determination in point-of-care diagnostics format. Electrochemical oxidation of doxorubicin on chemically modified electrodes makes it possible to detect its concentration on the level of n × 10^−8^ M [46]. This is insufficient for direct detection of the drug in blood, where subnanomolar concentrations should be detected.

Recently [25,26], we have shown that DNA could be used in a biosensor assembly as a collector of doxorubicin. Their interaction results in intercalation of the DNA helix followed by changes in its charge and volume. This can affect redox properties of the DNA carrier.

**Figure 6:**
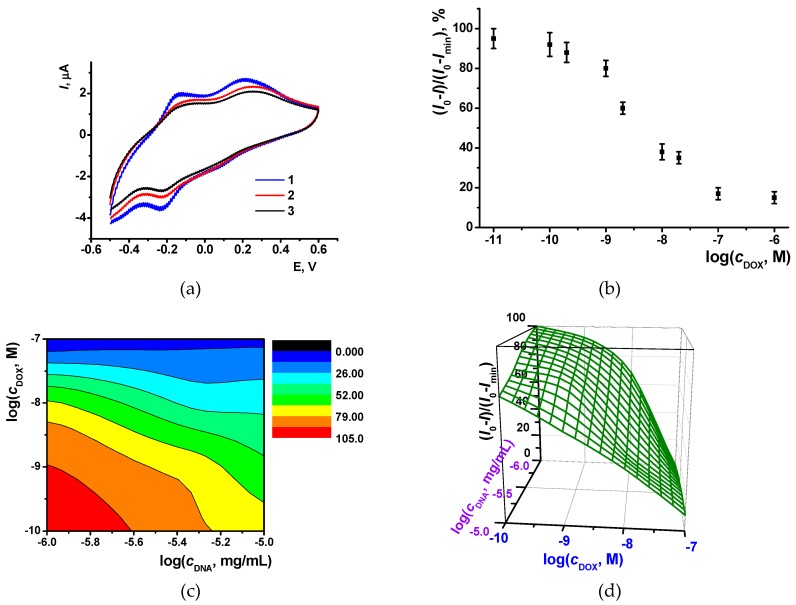
The influence of doxorubicin and DNA on the poly(Azure B) redox activity. Cyclic voltammograms recorded on GCE covered poly(Azure B) (1), mixture of 1 × 10^−5^ mg/mL DNA, and 1 × 10^−8^ M doxorubicin (2) with only 1 × 10^−5^ mg/mL DNA (**a**). Dependence of normalized response on doxorubicin concentration obtained for 1 × 10^−5^ mg/mL DNA (**b**) and 3D models obtained for normalized response measured at various DNA and doxorubicin concentrations (**c, d**).

Indeed, treatment of DNA with doxorubicin prior to its deposition on the electrode resulted in decrease of the peaks observed on poly(Azure B) modified GCE (Figure 6a). The stationary response was reached within 15 min. Quantitative analysis of doxorubicin has been performed using a normalized response (*I_0_ – I)/(I_0_ – I_min_*), where *I_0_* is the peak current recorded in the absence of doxorubicin and *I_min_* is the minimal peak current reached. The dependence of the normalized signal on the doxorubicin concentration *c* is described with logistic four-parameter model (4), where *a_1_* and *a_2_* are upper and lower limits of the dependency and $c_{0}$ and *p* are parameters characterizing the sensitivity of the analysis.

(4) $$\left(I_{0}-I\right)/\left(I_{0}-I_{\min}\right)=a_{2}+\frac{a_{1}-a_{2}}{1+{\left(\frac{c}{c_{0}}\right)}^{p}}$$

For DNA concentration equal to 1 × 10^−5^ mg/mL, *a*_1_ = 96 ± 4%, *a*_2_ = 13 ± 4%, $c_{0}$ = 3.7 ± 0.4 nM and *p* = 0.80 ± 0.07. The quantification area can be roughly estimated using EC_20_ and EC_80_ levels (concentrations corresponding to 20 and 80% of the signal variation). These were assessed as 0.65 and 21 nM, respectively. The limit of detection (LOD) was calculated from S/N = 3 ratio as 0.25 nM. The middle part of the curve can be fitted by the linear model with the following parameters (5). The linear concentration range varies from 1 × 10^−7^ to 1 × 10^−10^ M and LOD as 7 × 10^−11^ M.

(*I*_0_ − *I*)/(*I*_0_ − *I_min_*), % = (−126 ± 12) − (21 ± 2) × log(*c*, M), *R*^2^ = 0.9852, *n* = 6 (5)

The normalized response depends on both DNA and doxorubicin concentration. Decreasing DNA concentration from 1 × 10^−5^ to 1 × 10^−6^ mg/L DNA makes linear part of the curve narrower as is shown on 3D surface calculated from the experimental data (Figure 6c,d). This is quite unusual because the less receptor concentration, the higher the sensitivity of the response is expected. It can be explained by the possible influence of the DNA concentration on the surface coverage mentioned above. At low DNA loading, doxorubicin retains the ability to adsorb on naked poly(Azure B) film, whereas at higher DNA concentrations the polymer surface is mostly covered with the DNA molecules so that changes in the peak currents are due to changes in relative saturation of the DNA molecules with doxorubicin molecules. Besides, protonation of the amino group of doxorubicin partially neutralizes the charge of phosphate residues of the DNA backbone. As a result, signal changes in the direction opposite to that of DNA influence.

The sensitivity of the doxorubicin determination was higher or similar to that of other electrochemical sensors based on direct oxidation of doxorubicin on the electrode or its interaction with a specific aptamer. The comparison of analytical characteristics of sensors is presented in Table 2.

Only DNA sensors based on polyaniline showed similar or slightly higher sensitivity and broader concentration range. However, they operate at acidic pH required for redox activity of polyaniline matrix. This might be inconvenient for the application of biosensors in biological fluids.

### 3.4. Measurement Precision

Six individual sensors have been prepared using the same set of reagents and then their response toward 10 nM doxorubicin as a middle of the concentration range was tested. The DNA concentration was equal to 1 × 10^−6^ mg/mL (aliquot 2 μL). The poly(Azure B) layer was obtained with 40 cycles of the potential cycling. The sensor-to-sensor repeatability was found to be 3.8% with freshly prepared sensors and 5.5% after six weeks storage in dry conditions at 4 °C. For the same period of time, the absolute signal of poly(Azure B) oxidation reduction on voltammograms decreased by 10% of magnitude.

### 3.5. Selectivity and Real Sample Analysis

The signal of the developed DNA sensor was measured in the presence of 0.1 mM species that might have interfered with the doxorubicin measurements. The following compounds were tested: ascorbic and uric acids, acetaminophen, sulfamethoxazole, CaCl_2_, glucose, mannose, lactose, and mannitol. Two latter components have been used as stabilizers in commercial preparations of doxorubicin (Doxorubicin-LANS^®^ and Doxorubicin-Teva^®^). No significant influence on the relative peak current of poly(Azure B) was found, although ascorbic and uric acids slightly increased the cathodic peak current against the HEPES buffer. BSA did not affect the signal of poly(Azure B) prior to DNA deposition but could decrease the signal after DNA adsorption with no respect if doxorubicin was added or not. Maxima concentration of BSA, which did not influence the doxorubicin determination, was assessed as 4 mg/mL.

Spiked samples mimicking electrolyte content of the blood serum were prepared with the Ringer-Locke’s solution. The results obtained are presented in Table 3.

Besides, three samples of blood serum from healthy volunteers were taken and spiked with doxorubicin. Serum proteins were removed by sedimentation with methanol followed by centrifugation. The maximal concentration of organic solvent did not exceed 5 vol. %. Sample treatment protocol was adapted from HPLC determination of anthracycline drugs [53]. All the spiked samples were measured in triplicate and the recovery was found to be 80 ± 5, 85 ± 5, and 85 ± 4% for 1.0, 5.0, and 10 nM, respectively. As could be seen, deviation of the results was higher than that for artificial plasma probably due to higher contribution of serum proteins. The recovery is significantly different from 100%, meaning that some losses of the analyte are probably due to its adsorption on the serum proteins. Moreover, dilution of the serum can likely improve results, but this also depends on the current levels of the drug residues in the patient blood. Thus, DNA sensor developed showed satisfactory characteristics of real sample analysis.

## 4. Conclusions

In this work, polymeric form of Azure B has been for the first time applied for voltammetric determination of doxorubicin, an anthracycline cytostatic drug widely applied for chemotherapy of cancer diseases. Contrary to similar sensors utilizing polyaniline, all the steps of assembling DNA immobilization and doxorubicin incubation can be performed by physiological pH. This simplifies the operation of DNA sensors for real sample analysis. The assembling of the surface layer and entrapment of DNA were confirmed using EIS and SEM. The incubation of DNA in doxorubicin solution results in a partial decrease of its influence on intrinsic redox activity of the polymer. This was attributed to partial neutralization of the negative DNA charge and increase of the volume of biopolymer after the analyte intercalation. Although the measurement protocol assumes single use of the biosensor, simplicity of its assembling and rather high reproducibility of main operational and analytical characteristics makes it promising for preliminary control of doxorubicin pharmacokinetics in the point-of-care format. The applicability of the poly(Azure B) based DNA sensor was confirmed using testing drug formulations containing doxorubicin as well as samples mimicking blood electrolytes. Further, serum blood taken from healthy volunteers was spiked with the doxorubicin. The recovery of about 90% was found. Further improvement of the analysis results can be expected from additional dilution of the samples.

## Figures and Tables

**Table 1:** The dependency of the peak currents (*I_p_*, μA) on the scan rate (ν, V/s) for the poly(Azure B) peaks recorded on the glassy carbon electrode (GCE) modified with polymer; *n* is the number of experimental points.

Peak Potential, V	log (*I*, μA) = a + b × log (ν, V/s)			
	a	b	*R* ^2^	*n*
0.25	1.08 ± 0.06	1.00 ± 0.04	0.987	8
−0.09	0.89 ± 0.02	0.90 ± 0.01	0.998	9
−0.18	1.34 ± 0.06	1.13 ± 0.07	0.983	9

**Table 2:** Analytical characteristics of doxorubicin determination with electrochemical sensors and DNA sensors.

Modifier	Concentration Range	LOD, nM	Ref.
Multiwalled carbon nanotubes	0.09–7.36 µM	3	[24]
Carbon black, Cu nanoparticles, Nafion	0.46–5.1 µM	24	[46]
Ag nanoparticles, carbon dots on reduced graphene oxide	1.0 µM–10 nM	2	[47]
Multiwalled carbon nanotubes, poly(lysine)	2.5 nM–0.25 µM	1	[48]
Poly(arginine)	69 nM–1.08 µM	0.1	[49]
Poly(taurine), β-cyclodextrin and graphene quantum dots	0.086–3.45 μM	12	[50]
Aptamer against doxorubicin	31–125 nM	28 nM	[51]
Polyaniline, DNA	0.1 nM–0.2 mM	0.01	[25]
Poly(Neutral red), pillar [5]arene, DNA	0.01–100 µM	0.1	[52]
Poly(Azure B)	0.1 µM–0.3 nM	0.1	This work

**Table 3:** Determination of 10 nM doxorubicin in spiked samples mimicking serum and in commercial medications. Average ± S.D. for six individual sensors.

Sample	Media	(*I_0_ – I)/(I_0_ – I_min_*), %	Recovery, %
Doxorubicin (Sigma)	Standard solution in HEPES	78 ± 1	-
	+ 4 mg/mL BSA	78 ± 3	100
	+ 40 mg/mL BSA	73 ± 5	105
Doxorubicin (Sigma)	Ringer-Locke’s solution	77 ± 2	101
Doxorubicin-LANS ^®^	Ringer-Locke’s solution	77 ± 2	101
Doxorubicin-TEVA ^®^	Ringer-Locke’s solution	80 ± 3	97
